# High salt induced hypertension leads to cognitive defect

**DOI:** 10.18632/oncotarget.21326

**Published:** 2017-09-27

**Authors:** Cui-Ping Guo, Zhen Wei, Fang Huang, Min Qin, Xing Li, Yu-Man Wang, Qun Wang, Jian-Zhi Wang, Rong Liu, Bin Zhang, Hong-Lian Li, Xiao-Chuan Wang

**Affiliations:** ^1^ Department of Pathophysiology, School of Basic Medicine, Key Laboratory of Ministry of Education of China for Neurological Disorders, Tongji Medical College, Huazhong University of Science and Technology, Wuhan 430030, China; ^2^ Division of Neurodegenerative Disorders, Co-innovation Center of Neuroregeneration, Nantong University, Nantong, JS 226001, China; ^3^ Department of Genetics and Genomic Sciences, Icahn School of Medicine at Mount Sinai, New York, NY 10029, USA; ^4^ Department of Histology and Embryology, School of Basic Medicine, Key Laboratory of Ministry of Education of China for Neurological Disorders, Tongji Medical College, Huazhong University of Science and Technology, Wuhan 430030, China

**Keywords:** high salt diet, hypertension, cognitive defects, synaptogenesis, cerebral blood flow

## Abstract

Although increasing evidences suggest a relationship between hypertension and brain function for years, it is still unclear whether hypertension constitutes a risk factor for cognitive decline and its underlying mechanism. In the present study, an experimental animal model of hypertension simply by feeding rats with high salt diet was employed. We found that long-term high salt intake caused a marked increase of systolic blood pressure linked to a declined regional cerebral blood flow. Fear conditioning and morris water maze behavioral test revealed that high salt diet induced hippocampal dependent spatial reference memory deficits, while a decreased synaptogenesis without neuronal loss in hippocampus was observed in high salt treated rats. Furthermore, we found that high salt induced a decrease of intracellular calcium, which inactivated CaMK II and resulted in dephosphorylation of CREB at Ser133. These findings suggest a novel etiopathogenic mechanism of cognitive deficit induced by hypertension, which is initiated by high salt diet.

## INTRODUCTION

Hypertension, also known as high blood pressure, is a pathological process in which the blood pressure in the arteries is persistently elevated. High blood pressure usually does not cause symptoms. Long-term high blood pressure, however, is a major risk factor for coronary artery disease, stroke, heart failure, peripheral vascular disease, vision loss, and chronic kidney disease [1–4]. Recent researches have implied that hypertension may also play an important role in the development of cognitive impairment, vascular dementia, and Alzheimer's disease (AD)[5–7]. A longitudinal study from 4-year assessment found that the risk of cognitive impairment was increased 2.8 times in persons with hypertension [8]. In persons with hypertension, treatment with antihypertensive drug therapy had a 30% lower incidence of dementia than persons with hypertension not treated with antihypertensive drug therapy [9]. These studies strongly support that there is a tight relationship between blood pressure (BP) and cognitive function, and this connection is biologically complex and still not fully understood.

Neuroimaging has showed that high systolic blood pressure is associated with smaller regional and total brain volumes [10] and reductions in brain volume over time [11]. However, the high diastolic blood pressure does not affect brain volume [12]. Chronic hypertension disrupts cerebral autoregulation [13] and reduces resting cerebral blood flow [14]. Cerebral hypoperfusion may be associated with the dysfunction of synaptic proteins synthesis [15]. Hypertension leading to endothelial dysfunction disrupts the coordinated coupling among neurons, glia, and cerebral blood flow in the microvasculature [16]. It has been reported that high salt intake results in hypertension and causes endothelial dysfunction [17, 18]. We here investigate whether high salt intake induced hypertension leads to cognitive defects and its underlying mechanism. We observed a hippocampal dependent spatial memory deficit linked to loss of synaptic plasticity and a decrease of phosphorylated CREB in rats treated with long-term high salt diet.

## RESULTS

### Weight and general physical state

**Figure 1:**
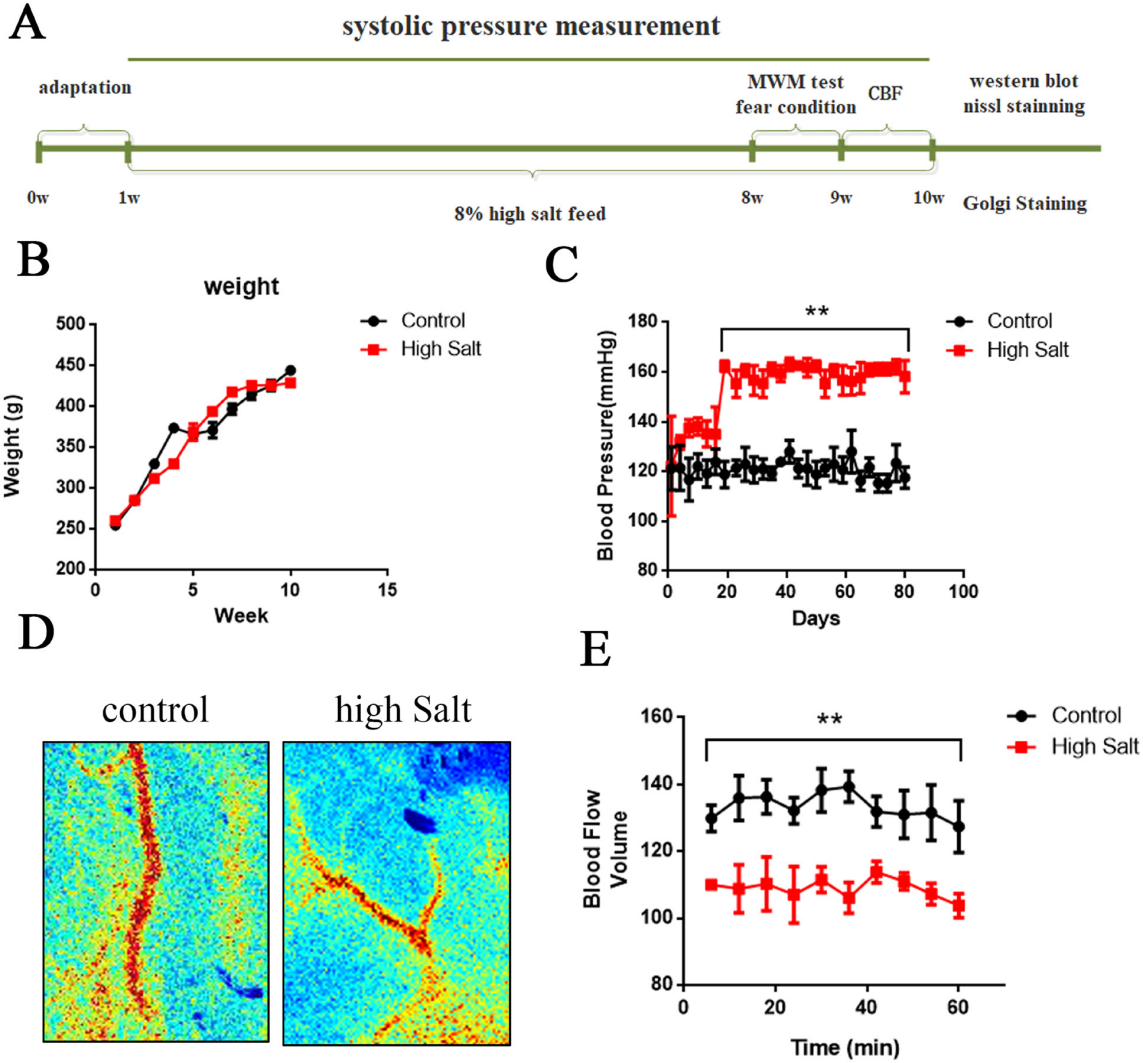
High salt diet leads to hypertension linked to a decrease of cerebral blood flow **(A)** Schematic representation of the outline of the study. **(B)** All rats were weighed once a week during the 9-weeks period of the study. **(C)** Using tail-cuff plethysmography to measure SBP, high salt diet markedly increased SBP compared to that of control rats with standard low-salt diet after 3 weeks high salt diet. **(D)** Cerebral blood flow was measured through the Laser Speckle Blood Flow Imager each ten minutes for one hour. **(E)** Quantitative analysis for blood flow of cerebral cortex. All data represent as mean ± SEM (n = 12). ^**^p < 0.01 versus control.

During the 9-weeks period of the study, animals were carefully checked and weighed once a week. We did not observe any alterations in general physical state, including grooming, posture, and clasping reflex, due to high salt diet. Statistical analysis did not reveal any significant group effect or group×week interaction in bodyweight (Figure 1B). These data suggested that high salt diet didn't induce significant effect on bodyweight among groups.

### Long-term high salt diet significantly raises systolic blood pressure and induces hypertension

High salt is a risk factor for hypertension [20, 21]. To establish the animal model of hypertension, we fed rats with a high salt diet (8% NaCl enriched diet) for 9 weeks. Using tail-cuff plethysmography to measure systolic blood pressure (SBP), rats fed with salt demonstrated a marked increase of SBP by 30±5 mmHg compared to that of control rats after 3 weeks high salt diet (Figure 1C). To test whether chronic hypertension may reduce resting cerebral blood flow in the present study, we employed the Laser Speckle Blood Flow Imager to detect the blood flow of cerebral cortex (Figure 1D). Quantitative analysis showed that high salt diet significantly decreased cerebral blood flow at each time point compared to control group (Figure 1E). Taken together, long-term high salt diet significantly raises systolic blood pressure and induces a chronic and sustained hypertension linked to a decrease of cerebral blood flow.

### High salt diet induced hypertension impairs spatial learning and memory in rats

**Figure 2:**
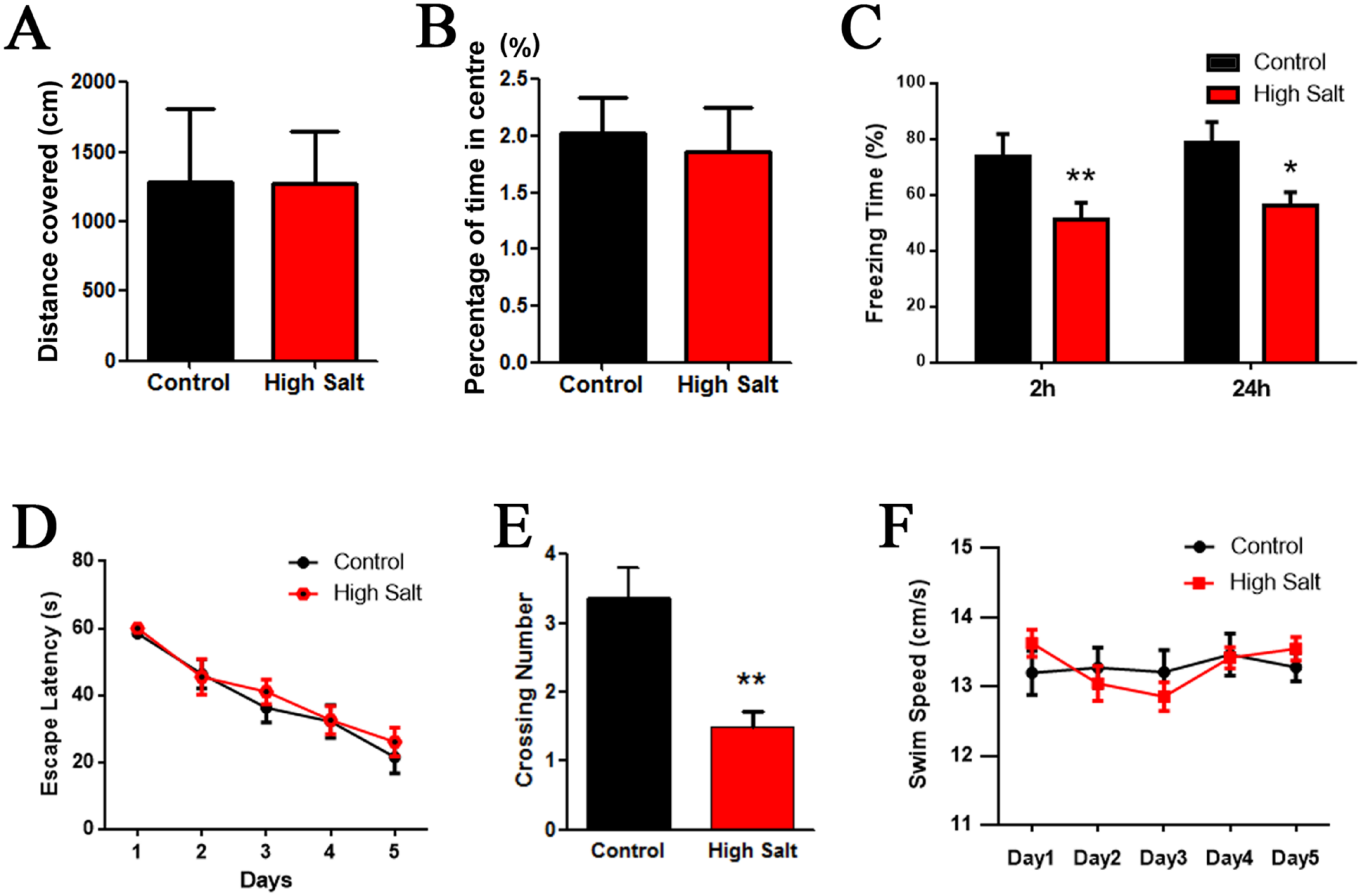
High salt induced hypertension induces cognitive impairment in rats **(A** and **B)** While, as compared with the control group, high salt rats showed no change in anxiety (A) or exploration (B) in the open field. **(C)** Fear conditioning was used to measure the contextual memory. After 2h and 24 h, the rats were put into the same training chamber without shocks, and the total freezing time in 3 min was recorded with a video camera. **(D)** Morris water maze test was employed to assess to learning and memory functions in the spatial reference memory task. Compared with the control group, the high salt rats showed no difference in finding the hidden platform compared to control groups during 5 days learning trials (D), while a marked decrease in the number of crossing the target quadrant during the transfer test **(E)**. **(F)** The mean swimming speed during the transfer test was no significant difference among each group. All data represent as mean ± SEM (n = 12). ^*^P < 0.05, ^**^P < 0.01 vs control.

To investigate whether the hypertension leads to cognitive deficit in rats with high salt diet, we tested rats in a classic spatial reference memory task highly involving the hippocampal integrity. Before the water-maze learning and memory test, anxiety and exploratory activity levels were evaluated in the open-field. Hypertension rats with high salt diet displayed similar patterns of anxiety and exploratory activity as control animals (Figure 2A and 2B). Next, we performed fear conditioning experiment to assess the contextual memory. We found that the rats with high saline diet exhibited significant lower freezing behavior compared to control (Figure 2C). Finally, we used MWM test to measure the effects of hypertension induced by high salt intake on hippocampus-dependent spatial learning and memory. No difference in finding the hidden platform was detected between high salt diet and the control groups during 5 days learning trials (Figure 2D). However, the latency to locate the target quadrate in high salt treated rats was significantly increased in the probe trials after removal of the platform compared to control group (Figure 2E), which confirms the impairment of spatial memory retrieval, as a possible result of hypertension. The swimming speed remained comparable among the groups, indicating that high salt diet does not affect motor function (Figure 2F). These data together demonstrate that high salt diet induced hypertension can cause hippocampus-dependent memory deficits.

### Loss of dendritic spines in the high salt-treated rats with hypertension

**Figure 3:**
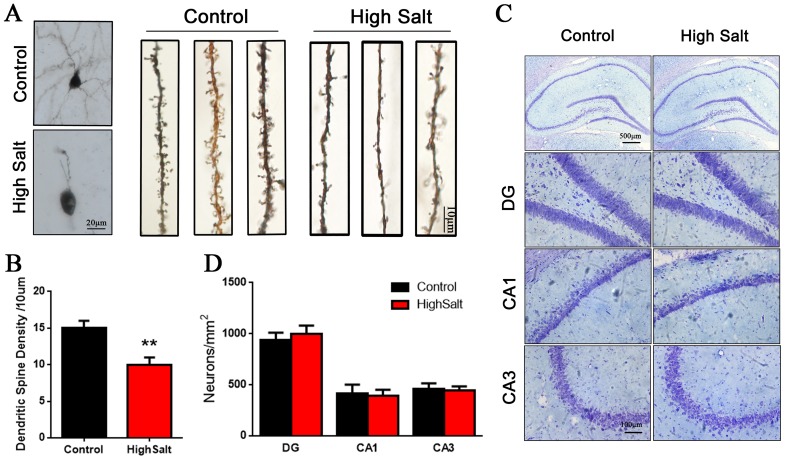
Loss of dendritic spines in the high saline-administrated rats **(A)** Golgi staining showed high salt rats display a decreased dendritic spine in hippocampus compared to wild type control rats. **(B)** Quantitative analysis for spine density from randomly selected dendritic segments of neurons. **(C)** Nissl staining was measured to the number of neurons in hippocampus. **(D)** Quantitative analysis for neuron density. All data represent as mean ± SEM, ^**^p < 0.01 versus control.

Previous studies have shown that chronic hypertension induces a decrease of cerebral blood flow, which may result in the dysfunction of synaptic proteins synthesis [5, 7]. To study the relationship between cognitive defects and synaptogenesis in the high salt-treated rats with hypertension, we employed Golgi staining and Nissl staining to detect the dendritic spines and the morphological structures of neurons. We found that hypertension rats displayed a marked decrease of dendritic spine density in hippocampus compared to control group (Figure 3A and 3B), while the number of neuron was no significant difference among the groups (Figure 3C and 3D). These data suggested that hypertension related cognitive defect is associated with loss of dendritic spines in hippocampus.

### High salt diet downregulates CaMKII/CREB pathway in rats linked to hypertension

**Figure 4:**
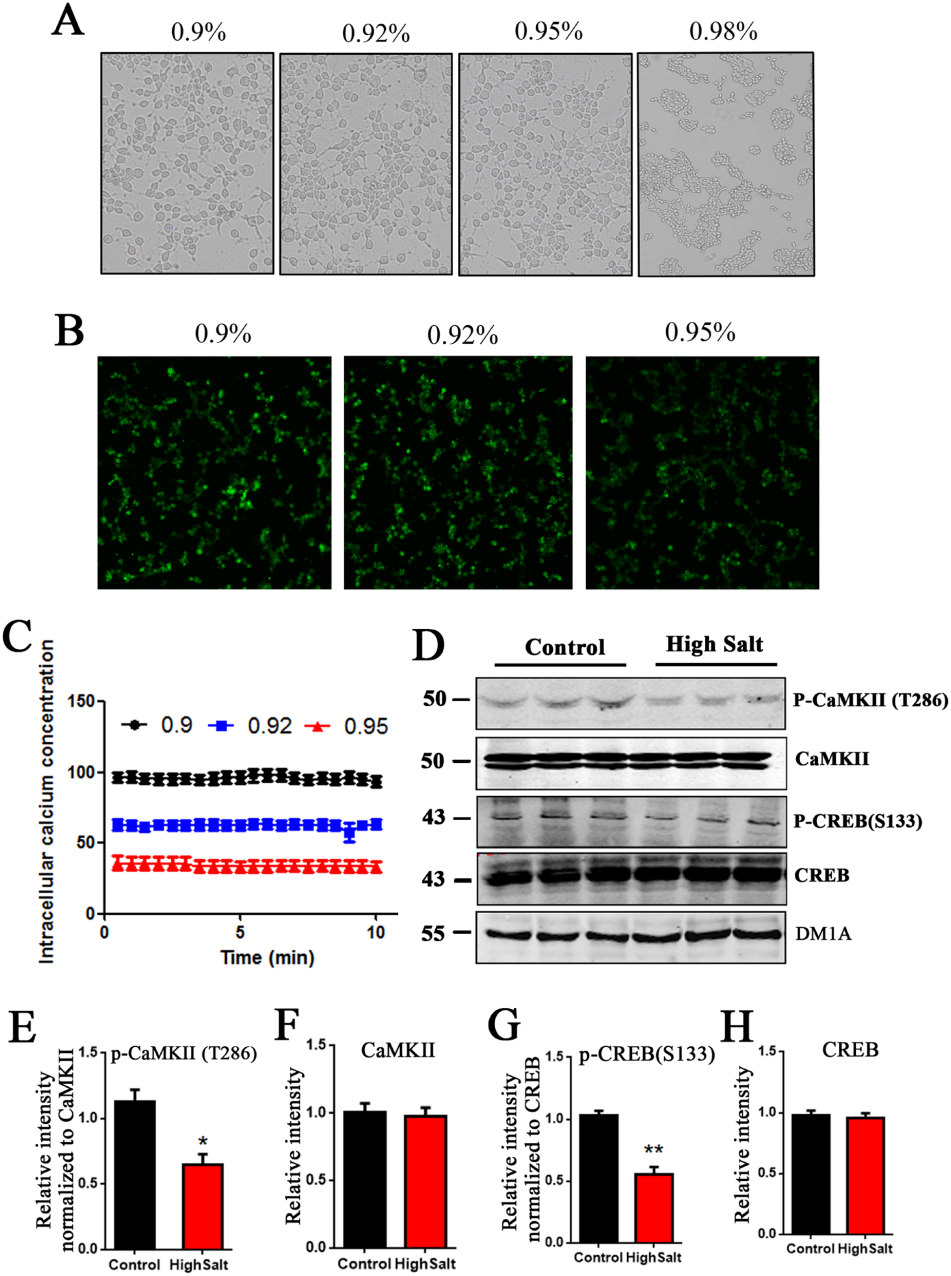
High salt diet induces a decreased [Ca2+]i and downregulates CaMKII/CREB pathway in rats **(A)** HEK293T cells were treated with 0.90%, 0.92%, 0.95%, 0.98% NaCl for 0.5 h at 37°C, and then cell morphology was recorded with bright field microscope imaging. **(B)** The calcium-sensitive indicator Fluo-3 AM treated cells by 2 μmol /L. The Fluo-3 signal was recorded by confocal recording. **(C)** Quantitative analysis for intracellular calcium concentration [Ca^2+^]_i_. **(D)** Western blots showed the phosphorylation and the total level of CaMKII and CREB in hippocampus, and **(E-H)** the quantitative analysis was performed. All data represent as mean ± SEM (n=3),^*^P < 0.05, ^**^P < 0.01 vs. control.

Sodium is an essential nutrient and the primary cation in the extracellular fluid [22, 23]. Previous study has shown that rats with high salt diet has higher sodium levels in the interstitial space [21]. To check the effect of high salt intake on intracellular calcium concentration, we used calcium-sensitive indicator Fluo-3 AM to measure intracellular calcium concentration [Ca^2+^]_i_ by fluorescence. Firstly, the HEK293T cells were exposed to various concentrations (0.9%, 0.92%,0.95%,0.98%) of NaCl for 0.5 h at 37°C. Compared with control group, the morphology of HEK293T cells treated with 0.9-0.95% NaCl have no obvious change. However, 0.98% NaCl induced cell shrinkage, but not filopodia or lamellipodia formation (Figure 4A). Considering the optimal living status of cells for experimentation, we chose 0.9-0.95% NaCl as the final concentration of salt administration in fluorescence assay with calcium dye (Figure 4B). Quantitative analyses showed that [Ca^2+^]_i_ was decreased in manner of increasing NaCl concentration (Figure 4C).

Some enzymes, including CaMKII, are calcium dependent. We here checked the CaMKII/CREB pathway, which is directed to learning and memory [24]. Western blots showed that high salt diet markedly decreased the expression of phosphorylated- CaMKII at (Thr286) in hippocampus of rats, but did not change the level of total CaMKII (Figure 4D, 4E and 4F). These data suggest that high salt intake leads to inactivation of CaMKII due to the decrease of intracellular calcium in hypertension rats. Accordingly, phosphorylation of CREB, a substrate of CaMKII, is also decreased (Figure 4D, 4G and 4H). Taken together, high salt diet downregulating CaMKII/CREB pathway may be involved in cognitive defects in hypertension rats.

### High salt diet doesn't induce Alzheimer like pathological alteration

**Figure 5:**
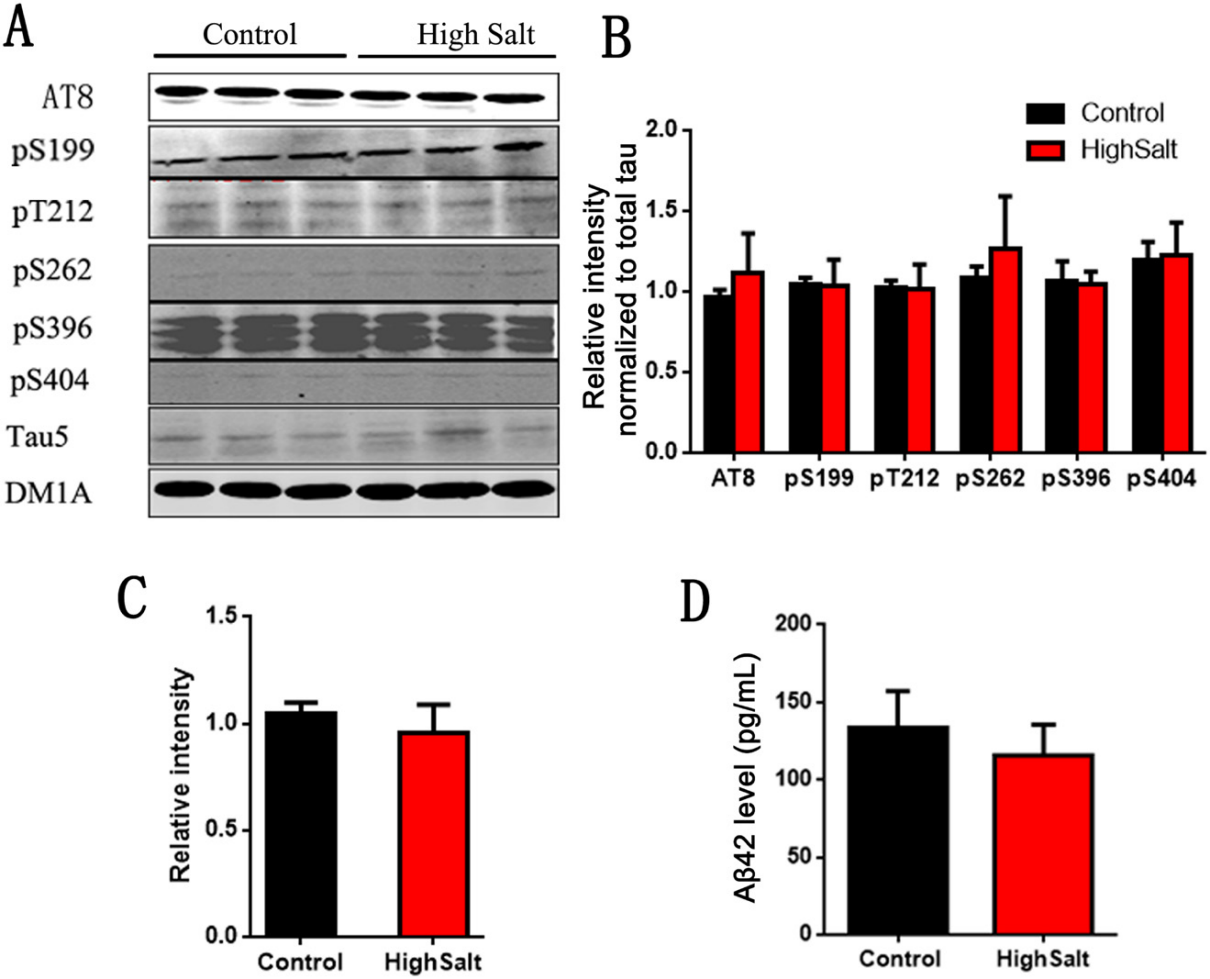
No change of Tau and Aβ in hippocampus of high salt diet **(A)** Western blots using AT8(Ser202/Thr205), pS199, pT212, pS262, pS396, pS404 and tau5 (total tau) showed tau phosphorylation and the level of total tau in hippocampus, and the quantitative analysis was performed **(B** and **C)**. **(D)** The Aβ_42_ levels in hippocampus were measured by ELISA kit. All data represent as mean ± SEM (n=3).

Hypertension has been reported to be a potential risk factor for Alzheimer's disease (AD)[25, 26]. To investigate whether high salt diet induced hypertension leads to AD like pathological changes, we detected its two hallmark lesions: extracellular senile plaques consisting of β-amyloid and intracellular neurofibrillary tangles made up of the abnormally hyperphosphorylated tau [27, 28]. To test the effect of high salt intake on tau phosphorylation, we here also detected the phosphorylation of tau at Ser199, Ser202/Thr205 (AT8), Thr212, Ser262, Ser396, Ser404 and total tau (Tau5) in rats (Figure 5A). Quantitative analyses showed that tau phosphorylation at above sites had no significant difference among groups (Figure 5B and 5C). We also quantitatively analyzed Aβ levels, and found that Aβ42 were no obvious difference among groups (Figure 5D). Thus, these data strongly support that high salt diet doesn't induce AD like pathological alteration.

## DISCUSSION

Here we have shown that 2 months old rats fed 8% NaCl enriched diet for 3 weeks appeared hypertension linked to a decreased regional cerebral blood flow. Moreover, lasting 7 weeks high salt diet induced hypertension leads to a hippocampal dependent memory deficit accompanied with loss of dendritic spines and downregulation of CaMKII/CREB pathway. In the present study, AD like pathological changes were not observed in high salt rats with hypertension. Together our data strongly support the conclusion that hypertension induced by long term high salt diet is a risk factor of cognitive defects.

A survey conducted in China have shown that the average salt intake is inversely proportion to blood pressure [29]. Actually, the average daily sodium intake in China is 10.6 g, 3 times the recommended by the WHO. Therefore, high salt intake might be an important risk factor for hypertension in China [30–32]. Recent study showed that a 12 weeks of chronic 0.9% sodium diet intake after weaning leads to hypertension in adult rats [33]. We here employed a high salt diet (8% NaCl enriched diet) to rats, and found that only 3 weeks of 8% sodium diet intake leads to hypertension in adult rats accompanied with a decrease of cerebral blood flow. Hypertension can induce a change in arteriole endothelial and vascular smooth muscle cells that diminish cerebrovascular reactivity [34], which in turn shifts the cerebral autoregulatory curve and reduces resting cerebral blood flow [14].

Accumulating data implies that hypertension may be involved in development of cognitive impairment [5–7]. However, the underlying mechanism of hypertension related cognitive defects remain still unclear. Some researchers have shown that hypertension associated with smaller regional and total brain volumes, decrease of cerebral blood flow, white matter microstructural damage, vascular oxidative stress and cerebral small vessel disease might be implicated to cognitive impairment [35, 36]. In the present study, we found long term high salt diet induces a hippocampal dependent memory deficit in hypertension rats, which accompany with a decreased synaptogenesis without neuronal loss in hippocampus. It is consistent with previous study showing that oligemia may downregulate the synthesis of proteins necessary for synaptic plasticity and memory formation [15]. Meanwhile, a memory related pathway, CaMKII/CREB signals is downregulated in hypertension rats with high intake. Due to a higher sodium levels in the interstitial space under high salt diet condition [37], we suppose that high sodium stimulates sodium-calcium exchanger to remove calcium from cells [38]. We found that high sodium induces a decrease of intracellular calcium, which in turn inactivates CaMKII and results in dephosphorylation of CREB at Ser133, finally downregulates CaMKII/CREB pathway and leads to cognitive impairment.

Treatment of hypertension may reduce cognitive deficit and Alzheimer's disease (AD)[39, 40]. However, the data from randomized, controlled clinical trials for antihypertensive drug treatment preventing dementia are conflicting [40]. AD is characterized by the presence of two hallmark lesions: extracellular senile plaques consisting of β-amyloid and intracellular neurofibrillary tangles made up of the abnormally hyperphosphorylated tau [41–43]. In the present study, we didn't observe the increase of tau phosphorylation and Aβ in hypertension rats treated with high salt. It might be because the high salt diet is not enough long or salt induced hypertension is not sensitive to AD pathological process.

In summary, our results further confirm that a relative long term high salt intake induces a chronic and sustained hypertension linked to a decrease of cerebral blood flow. Together with loss of dendritic spines and CaMKII/CREB pathway dysfunction results in cognitive defects in hypertension rats. We here report a novel etiopathogenic mechanism of cognitive impairment related to hypertension, which is initiated by high salt diet.

## MATERIALS AND METHODS

### Chemicals and antibodies

Bicinchoninic acid (BCA) protein detection kit was from Pierce (Rockford, IL, USA). Reagents for cell culture were from Gibco BRL (Gaithersburg, MD, USA). Antibodies employed in this study are listed in Table 1.

**Table 1:** Antibodies employed in this study

Antibody	Specific	Type	Dilution	Source
p-CaMK II	Phosphorylated at T286	Poly-	1:500 for WB	Cell Signaling Danvers, MA, USA
CaMK II	Total CaMK II	Mono-	1:1000 for WB	Cell Signaling Danvers, MA, USA
P-CREB	Phosphorylated at S133	Poly-	1:500 for WB	Cell Signaling Danvers, MA, USA
CREB	Total CREB	Mono-	1:1000 for WB	Cell Signaling Danvers, MA, USA
Tau5	Total tau	Mono-	1:1000 for WB	MerckMillipore, Germany
AT8	Phosphorylated at Ser202 and Thr205	Mono-	1:1000 for WB	Thermo Fisher Scientific, USA
pS199	Phosphorylated at S199	Poly-	1:500 for WB	Thermo Fisher Scientific, USA
pT212	Phosphorylated at Thr212	Poly-	1:500 for WB	United Chemi-con, USA
pS262	Phosphorylated at S262	Poly-	1:500 for WB	Singnalway Antibody, USA
pS396	Phosphorylated at S396	Poly-	1:1000 for WB	Singnalway Antibody, USA
pS404	Phosphorylated at S404	Poly-	1:500 for WB	Singnalway Antibody, USA
DMIA	Total DM1A	Mono-	1:1000 for WB	Cell Signaling Danvers, MA, USA

Mono-, monoclonal; poly-, polyclonal; WB, Western blotting.

### Animals

The male Sprague-Dawley rats (2 months old, 250± 20 g) were supplied by the Experimental Animal Central of Tongji Medical College, Huazhong University of Science and Technology. After one week of acclimatization, rats were randomly divided in two groups: (i) 12 rats were fed with a standard low-salt diet (0.26% NaCl enriched diet) as a control and (ii) 12 rats were fed with a high-salt diet (8% NaClenriched diet) for 9 weeks [19]. The rats were housed with free access to food and water under a 12:12-hour reversed light-dark cycle, with light on at 8:00 PM. All animal experiments were performed according to the “Policies on the Use of Animals and Humans in Neuroscience Research” revised and approved by the Society for Neuroscience in 1995, and the animal study was approved by the Academic Review Board of Tongji Medical College. After 7 weeks of high salt diet, animals were first submitted to cognitive tests, and then cerebral blood flow, morris water maze, nissl staining, golgi staining, western blotting, immunohistochemistry tests were carried out (Figure 1A).

### Blood pressure measurement

Noninvasive BP was measured using a tail-cuff system. Briefly, all rats were held in a restrainer on a warmer for 10 min, a cuff was attached to the tail and BP was then recorded. The systolic blood pressure (SBP) was obtained as an average of three measurements. Each rat was trained to become familiar with this procedure for 3 days and also received the BP measurement as the self-control. The SBP was measured after high saline diet from the second week to the ninth week.

### Cerebral blood flow measurement

The rats were anesthetized with 10% chloral hydrate by intraperitoneal injection of 0.5 ml/100g. After anesthesia, the rats were fixed in rat stereotaxic device, and the anterior fontanel and lateral oculi were fully exposed with separation of subcutaneous connective tissue. And then, dental drill (0.5mm) was employed to open the cranial window and remove the parietal bone. Next, the rats were placed into the laser patch-like imaging system (Britton Chance center for biomedical photonics, Wuhan) while the brain tissue in physiological status and the smooth surface of the image were kept. Using 650nm red laser irradiation, the regional cerebral blood flow is recorded under exposure time of 40 milliseconds, frame interval of 1000 milliseconds, recording 60 minutes.

### Open-field activity

Anxiety and exploratory activity were evaluated by allowing rats to freely explore an open field for 20 min. The testing apparatus was a classic open field (i.e., a PVC square arena of 100×100 cm^2^, with 70-cm-high walls). The open field was placed in a part of the room separated from the experimenter and the control station with a black opaque curtain. Rats were individually submitted to a single 20-min session. Because for rodents the middle of a non-familiar arena is anxiogenic, anxiety was studied by analyzing the percentage of time spent in the middle of the arena. To assess exploratory activity, the total distance that the animals covered in the arena was tracked and measured. Data collection was performed using tracking files of the experiment recorded with LabState ver 1.0 software (AniLab Software & Instruments Co., Ltd, China).

### Fear conditioning

To test the effect of spatial training on the hippocampus-dependent associative memory. Rats were placed into a square chamber with a grid floor. On the first day, each rat was habituated to the chamber for 3 min, and then an auditory cue was delivered (70 dB, 30 s) followed by a foot shock (0.5 mA, 2 s). The same procedure was repeated two times with 2-min intervals. After 2 h, the rats were put into the same chamber for without any stimulus for 3 min, and freezing time was recorded for assessment of memory. On the next day, the rats were exposed to the same chamber without any stimulus for 3 min. The contextual conditioning was assessed by recording freezing behavior.

### Morris water maze (MWM)

The standard MWM procedure with minor modifications was used for the spatial training. For spatial learning, the rats were trained in the water maze to find a hidden platform for 5 consecutive days, four trials per day (with a 40-min interval) from 8:00 AM to 14:00 AM. In each trial, the rat started from one of four quadrants facing the wall of the pool and ended when the animal climbed on the platform. If the rat did not locate the platform in 60 s, they were guided to the platform and to stay in the platform for 20 s uniformly. The swimming path and the time used to find the platform (latency) was recorded by a video camera. Spatial memory was tested after training. The platform was removed and the percentage of time spent in the target quadrant and the number of platform crossings were recorded.

### Golgi staining

After the last BP measurement, rats were anesthetized with 6% chloral hydrate (0.6ml/kg, i.p.) and transcardially perfused with proximately 300 ml of normal saline containing 0.5% sodium nitrite, followed with 300 ml of 4% formaldehyde solution and then 500ml staining solution (5% chloral hydrate, 5% potassium dichromate and 4% formaldehyde) over 2 hours. Brains were removed from the skull and incubated in the staining solution for 3 days and in 1% silver nitrate solution for another 3 days in the dark. The silver nitrate solution was changed every day. Finally, the brains were sliced using a vibrate microtome (Leica, Wetzlar, Germany) at a thickness of 80μm.

### Nissl staining

The slice was pasted on the slide and dried by airing for 24 h in room temperature. Use the toluidine blue (Wuhan Goodbio technology CO, LTD, China) to stain for1 min and wash by PBS for 10 min with three times. According to the color, use the 95% ethanol to decolorize for 3 min. Finally, use the absolute ethyl alcohol for dehydration for 5 min three times and the dimethylbenzene for transparency for 10 min twice. Image-Pro Plus software was used to analyze cell numbers. The areas covered in regions of the hippocampus were measured. Three to five coronal sections were analyzed per rat, and the average ratios were used to calculate group means.

### Measurement of intracellular calcium concentration

Fura-3-acetoxymethyl ester (Fura-3-AM) is a membrane-permeable derivative of the ratiometric calcium indicator Fura-3 used in biochemistry to measure intracellular calcium concentrations [Ca^2+^]_i_ by fluorescence. In this study, HEK293T cells were treated with 0.9%, 0.92%, 0.95% 0.98% NaCl solution. The cells were incubated for 30 minutes, grown in a humid atmosphere containing 5% CO_2_ at 37°C. Cells were then incubated with final concentration of 2μMFura-3-AM dissolved in dimethyl sulphoxide (DMSO) in the dark for 30 min, and washed cells three times with standard extra cellular fluid mentioned above in the dark, and then using confocal microscopy (Olympus, Tokyo, Japan) and analysis software to measure the intensity of fluorescence of randomly chosen cells which showed clear cell configuration.

### Western blotting

The hippocampal tissues were rinsed twice in ice-cold PBS (pH 7.5) and lysed with buffer containing 10 mM Tris-Cl, pH 7.6, 50 mM NaF, 1 mM Na_3_VO_4_, 1 mM edetic acid, 1 mM benzamidine, 1 mM PMSF, and a mixture of aprotinin, leupeptin, and pepstatin A (10μg/ml each). After measurement of protein concentration in the extracts using BCA kit (Pierce, Rockford, IL), a final concentration of 10% β-mercaptoethanol and 0.05% bromophenol blue were added, and the samples were boiled for 10 min in a water bath for Western blotting. The proteins in the extracts were separated by 10% SDS-PAGE and transferred to nitrocellulose membrane. The membrane was then blocked in 5% non-fat milk for 1 hour at room temperature. The membranes were incubated with primary antibody at 4 °C for overnight. Then the blots were incubated with anti-rabbit or anti-Goat IgG conjugated to IRDye™ (800CW) (1:10000) for 1 h at room temperature. Immunoreactive bands were visualized using the Odyssey Infrared Imaging System (Licor biosciences, Lincoln, NE). To eliminate the variations due to protein quantity and quality, the data were adjusted to DM1A expression (IOD of objective protein *versus* IOD of DM1A protein).

### ELISA quantification of Aβ

To detect the concentration of Aβin hippocampus, the rat hippocampus were homogenized in buffer (PBS with 5% BSA and 0.03%Tween-20, supplemented with protease inhibitor cocktail), and centrifuged at16,000g for 20 min. Aβ1-42 was quantified using the rat Aβ1-42 ELISA Kit (Elabscience, Wuhan, China) in accordance with the manufacturer's instructions. The Aβ concentrations were determined by comparison with the standard curve.

### Statistical analysis

All experiments were repeated at least three times. Experimental values were obtained from three independent experiments with a similar pattern and expressed as means. Data were expressed as mean ± SEM and analyzed using SPSS 18.0 statistical software (SPSS Inc, Chicago, Illinois, USA). For analysis involving multiple groups, ANOVA procedure followed by Student-Newman-Keuls post hoc test with 95% confidence and Student's two-tailed t-test. Differences with p<0.05were considered significant.

## Abbreviations

AD: Alzheimer's disease
BP: blood pressure
SBP: systolic blood pressure
CBF: cerebral blood flow
